# Clinical Factors and Quantitative CT Parameters Associated With ICU Admission in Patients of COVID-19 Pneumonia: A Multicenter Study

**DOI:** 10.3389/fpubh.2021.648360

**Published:** 2021-04-22

**Authors:** Chengxi Yan, Ying Chang, Huan Yu, Jingxu Xu, Chencui Huang, Minglei Yang, Yiqiao Wang, Di Wang, Tian Yu, Shuqin Wei, Zhenyu Li, Feifei Gong, Mingqing Kou, Wenjing Gou, Qili Zhao, Penghui Sun, Xiuqin Jia, Zhaoyang Fan, Jiali Xu, Sijie Li, Qi Yang

**Affiliations:** ^1^Xuanwu Hospital, Capital Medical University, Beijing, China; ^2^Liangxiang Teaching Hospital, Capital Medical University, Beijing, China; ^3^Department of Research Collaboration, R&D Center, Beijing Deepwise and League of PHD Technology Co., Ltd., Beijing, China; ^4^Neusoft Institute of Intelligent Healthcare Technology, Beijing, China; ^5^The Second Affiliated Hospital of Harbin Medical University, Harbin, China; ^6^The Third Central Hospital of Tianjin, Tianjin, China; ^7^Sixth People's Hospital of Xinjiang Autonomous Region, Xinjiang, China; ^8^Central Hospital Hongxinglong Administration Bureau Youyi County, Shuangyashan, China; ^9^Central Hospital Affiliated to Xinxiang Medical University, Xinxiang, China; ^10^Harbin Chest Hospital, Harbin, China; ^11^Shanxi Provincial People's Hospital, Taiyuan, China; ^12^Sichuan Provincial People's Hospital, Chengdu, China; ^13^Langfang People's Hospital, Hebei, China; ^14^Beijing Chaoyang Hospital, Capital Medical University, Beijing, China; ^15^Radiology, Keck School of Medicine, University of Southern California, Los Angeles, CA, United States

**Keywords:** COVID-19, computed tomography, intensive care unit, deep learning, pneumonia

## Abstract

The clinical spectrum of COVID-19 pneumonia is varied. Thus, it is important to identify risk factors at an early stage for predicting deterioration that require transferring the patients to ICU. A retrospective multicenter study was conducted on COVID-19 patients admitted to designated hospitals in China from Jan 17, 2020, to Feb 17, 2020. Clinical presentation, laboratory data, and quantitative CT parameters were also collected. The result showed that increasing risks of ICU admission were associated with age > 60 years (odds ratio [OR], 12.72; 95% confidence interval [CI], 2.42–24.61; *P* = 0.032), coexisting conditions (OR, 5.55; 95% CI, 1.59–19.38; *P* = 0.007) and CT derived total opacity percentage (TOP) (OR, 8.0; 95% CI, 1.45–39.29; *P* = 0.016). In conclusion, older age, coexisting conditions, larger TOP at the time of hospital admission are associated with ICU admission in patients with COVID-19 pneumonia. Early monitoring the progression of the disease and implementing appropriate therapies are warranted.

## Introduction

In December 2019, coronavirus disease 2019 (COVID-19) was identified in Wuhan, China (1). Its causative agent, the severe acute respiratory syndrome coronavirus 2 (SARS-CoV-2), was subsequently confirmed to have the same genus of subfamily orthocoronavirinae with the family coronaviridae (2). Thus, the virus exhibits a strong affinity to human respiratory receptors and commonly causes lower-respiratory tract infection (3, 4). According to the World Health Organization, the most common diagnosis for severe COVID-19 is severe pneumonia (5). In China, it was estimated that 15–20% of people infected with COVID-19 developed severe pneumonia and 5–10% required admission to intensive care unit (ICU) (6). Previous studies demonstrated the imaging findings and clinical presentations in patients with COVID-19 pneumonia (7–10). The clinical spectrum of COVID-19 pneumonia ranges from mild to critically ill cases (1). Although most patients with mild symptoms have good outcomes, those who have been admitted to ICU can experience acute respiratory distress syndrome (ARDS), acute kidney injury, multiple organ failure with a considerably high mortality rate (11–13).

Computed tomography (CT) played an important role in the diagnosis and management of COVID-19 pneumonia. Compared with real-time reverse transcription-polymerase chain reaction assay (r-RT-PCR), CT has not been widely recommended as a first-line imaging modality yet for diagnosis due to its high sensitivity but limited specificity (14). Typical CT findings (such as bilateral and subpleural areas of ground-glass opacification (GGO), consolidation affecting the lower lobes) may help early ascertain virus pneumonia and further helped evaluate the extent of severity of COVID-19 pneumonia (9, 15, 16). However, accurate evaluation of chest CT images still depends on the radiologist's experience and is often qualitative.

Artificial intelligence (AI) technology has been shown to have the ability to help make important treatment decisions in urgent settings based on multiple factors (13). Imaging findings were important components in training dataset built for predicting acute lung injury (13, 17). Quantitative analysis of lung lesions on chest CT images using AI models was shown to improve chest CT interpretation for COVID-19 pneumonia (18–20). Previous studies assessed risk factors associated with adverse composite endpoints in COVID-19, however, they included clinical or quantitative CT information alone, respectively (18, 19). We hypothesized that combined quantitative parameters originated from chest CT and clinical parameters on hospital admission would help to predict the risk of ICU admission in COVID-19 pneumonia patient.

## Methods

### Data Collection

**Figure 1:**
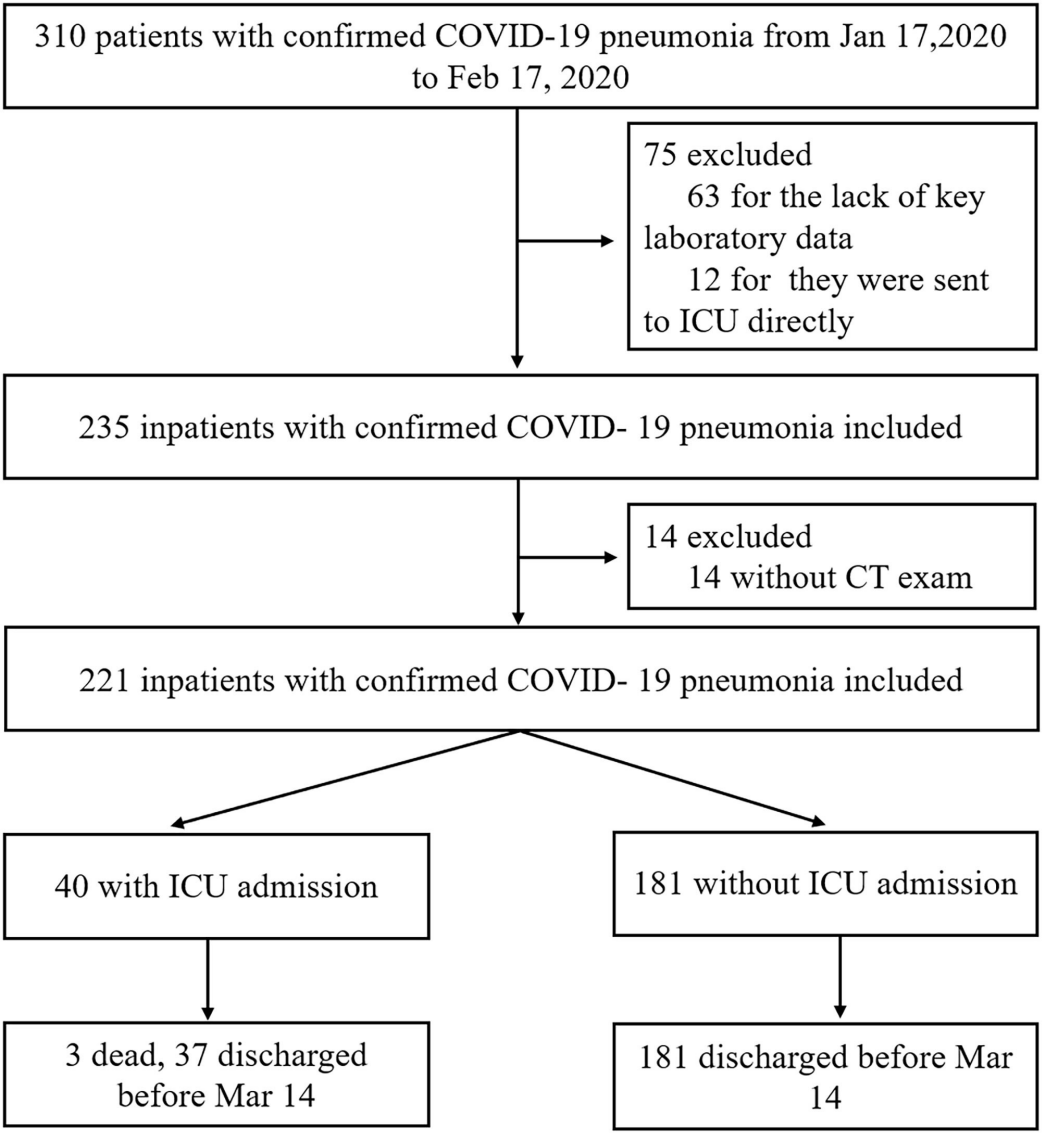
Study flow diagram.

A total of 310 consecutive patients who were admitted to 10 designated hospitals in China from January 17, 2020 to February 17, 2020 for a confirmed diagnosis of COVID-19 pneumonia were retrospectively enrolled. All patients were consecutively included during the study period. Inclusion criteria were as follows: (1) real-time reverse-transcriptase polymerase-chain-reaction detection of SARS-CoV-2 nucleic acid positive in throat swabs or lower respiratory tract; (2) the virus gene sequencing of respiratory or blood samples was highly homologous with SARS-CoV-2; (3) having underwent CT examination on admission. Exclusion criteria were as follows: (1) patients without CT examination on admission; (2) lack of key laboratory data; (3) patients were sent to ICU directly. By reviewing and analyzing admission logs and histories from all available electronic medical records and patient care resources, patients who were later transferred to ICU were identified. For patients who were alive by February 17, their living status was confirmed by March 14. Patient age, gender, exposure history, coexisting conditions [cardiovascular disease (CVD), chronic obstructive pulmonary disease (COPD), chronic liver disease, diabetes mellitus (DM)], onset symptoms, vital signs, and laboratory test findings (blood routine test, biochemical indices of kidney and liver, Inflammatory marker, and so on) were collected on admission from all participating hospitals. Finally, 221 patients were enrolled in our study. Among those patients, 89 were excluded—including 63 patients without key laboratory data, 12 patients directly admitted to ICU, and 14 patients with only chest X-ray examinations during hospitalization. During the follow-up, 40 patients (18%) were transferred to ICU (3/40 cases of mortality before Mar 14) because they required high-flow nasal cannula or higher-level oxygen support measures to correct hypoxaemia (21); 181 patients (82%) were not transferred to ICU. 218 patients were discharged, as shown in Figure 1. Ethical approval was granted by the Chaoyang Hospital Ethics Committee (2020-science-26).

### Imaging Technique and Image Interpretation

**Figure 2:**
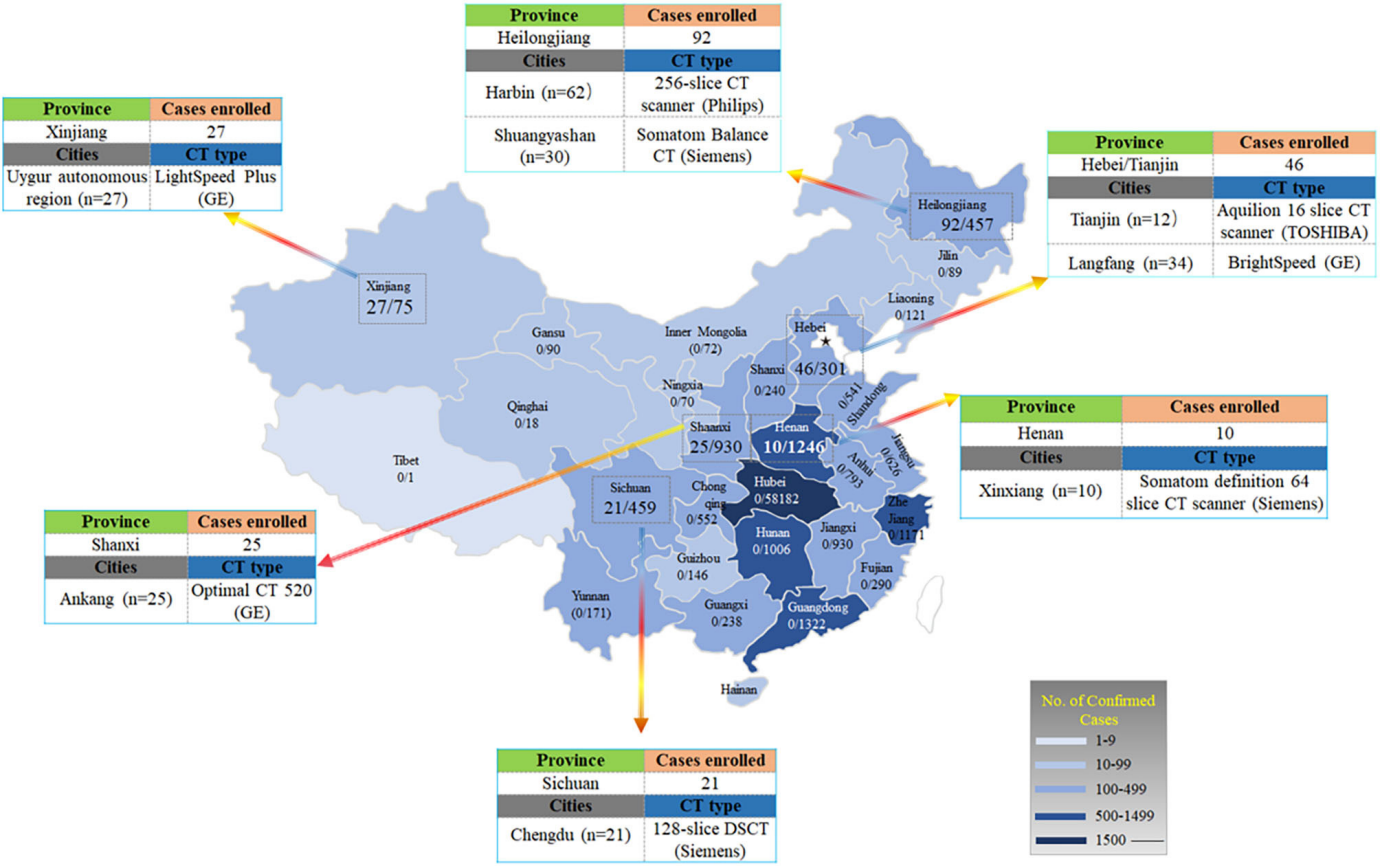
Distribution of patients with COVID-19 across China. It shows the official statistics of all documented, laboratory-confirmed cases of COVID-19 throughout China, according to the National Health Commission as of February 17, 2020. The numerator denotes the number of patients who were included in the study cohort and the denominator denotes the number of laboratory-confirmed cases for each province or autonomous region, as reported by the National Health Commission.

As shown in Figure 2, 62 patients from Harbin (Heilongjiang Province) were scanned using a 256-slice CT scanner (Philips Healthcare, Cleveland, OH, US), 30 patients from Shuangyashan (Heilongjiang Province) were scanned with a Somatom Balance CT scanner (Siemens Healthcare, Forchheim, Germany), 27 patients from the Uygur autonomous region (Xinjiang Province) were examined with LightSpeed Plus (GE, Medical System, Milwaukee, USA), 21 patients from Chengdu (Sichuan Province) were examined with a 128-slice dual-source CT (Siemens Healthcare, Forchheim, Germany), and 10 patients from Xinxiang (Henan Province) were imaged with a Somatom definition 64 slice CT scanner (Siemens Healthcare, Forchheim, Germany). Twenty-five patients from Ankang (Shaanxi Province) were imaged with 16 slice, Optimal CT 520 (GE, Medical System, Milwaukee, USA), 34 patients from Langfang (Hebei Province) were imaged with BrightSpeed (GE, Medical System, Milwaukee, USA), and 12 patients from Tianjin (Hebei Province) were imaged with Aquilion 16 slice CT scanner (TOSHIBA, Medical Systems, Tokyo, Japan). All these CT images were reconstructed into a slice thickness of 1.0–5.0 mm. Scan were performed in the supine position during end-inspiration.

### Lung Lesion and Lung Lobe Segmentation

**Figure 3:**
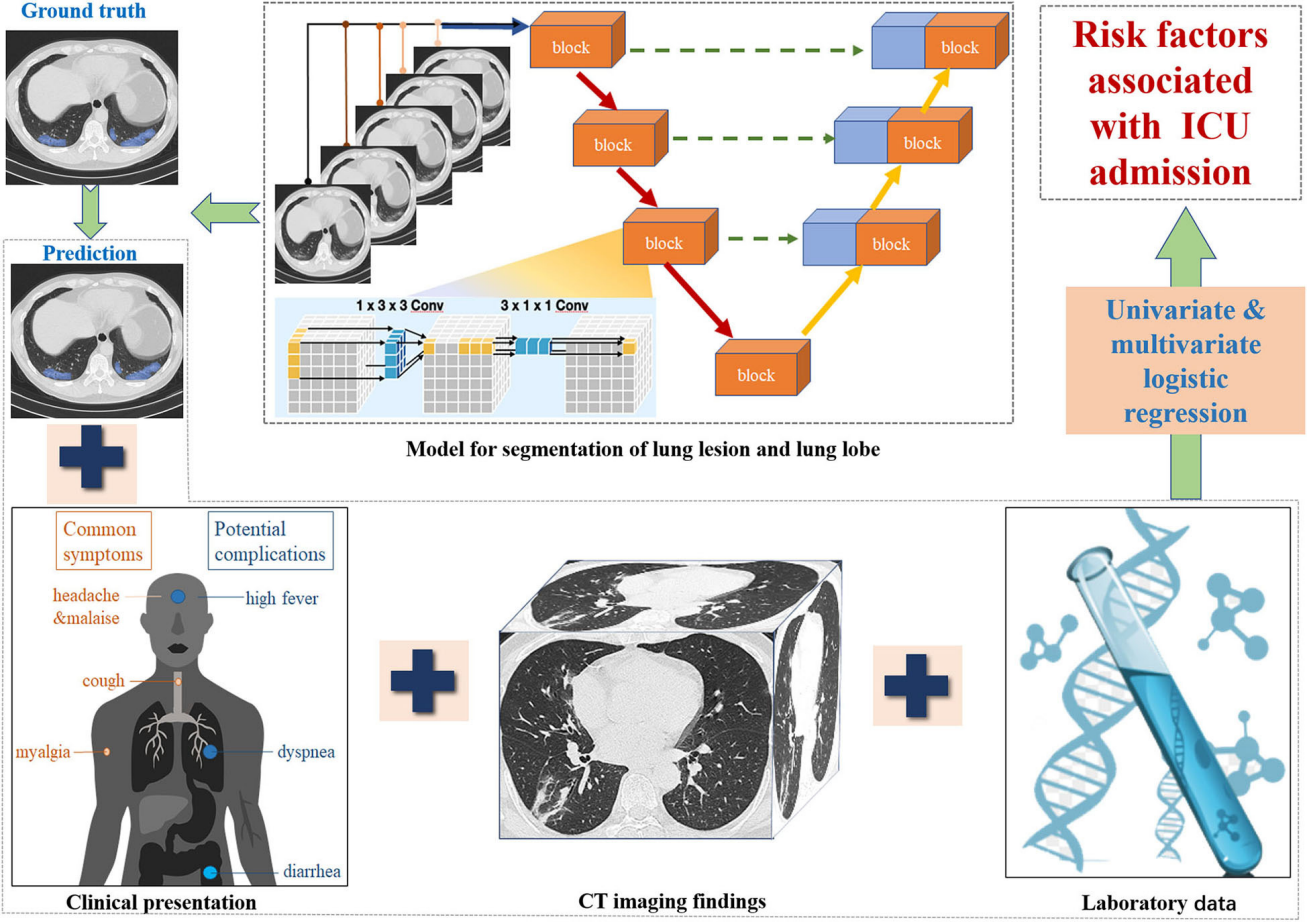
Workflow of ICU prediction in COVID-19 pneumonia.

In order to analyze lung lesions quantitatively, a deep learning model was used to segment lung lobes and lesions. All the chest CT images were anonymized and evaluated by a DL-based computer-aided diagnostic system (Dr. Wise Multimodal Research Platform, Deepwise, Beijing, China), which was trained with CT scans of patients with pulmonary disease (22). The overall framework of our proposed lung lobe and lung infection segmentation algorithm is shown in Figure 3. Our proposed segmentation network was a U-Net based architecture which employs pseudo 3D convolution as its building blocks (See the Supplementary Materials).

Then, we calculated quantitative parameters including total opacity percentage (TOP), total opacity volume (TOV), total GGO percentage (TGP), total GGO volume (TGV), total consolidation percentage (TCP), and total consolidation volume (TCV). TOV, TGV, and TCV are computed by product of the number of voxels in the lesion region and the volume of each voxel. TOP, TGP, and TCP were defined as following:

(1) $$\begin{array}{r} TOP\;=\;\frac{TOV}{TLV} \end{array}$$

(2) $$\begin{array}{r} TGP\;=\;\frac{TGV}{TLV} \end{array}$$

(3) $$\begin{array}{r} TCP\;=\;\frac{TCV}{TLV} \end{array}$$

where TLV means the total volume of lung region.

### Expert Interpretation

Seven imaging features were assessed using international standard terms defined by Fleischner Society glossary and peer-reviewed literatures on viral pneumonia, including GGO, consolidation, nodule, linear opacities, reticular pattern, interlobular septal thickening, and mixed pattern (23). Extrapulmonary abnormalities, including pleural effusion, lymph node with the short axis exceeding 10 mm, coronary calcification, and aortic calcification, were also recorded. Two radiologists (Y.C.X and Y.H) with the experience of 5 and 10 years performed the consensus blind review. The involvement degree of each lung lobe was classified as the score of 0 (0% involvement), 1 (<25% involvement), 2 (25 to <50% involvement), 3 (50 to <75% involvement), or 4 (75% or greater involvement). By summing up the five lobe scores, overall lung severity was recorded as a CT score (15). These images were firstly assessed by a middle-aged radiologist and then reviewed and revised by senior radiologist.

### Statistical Analysis

Statistical analysis was performed using SPSS version 21.0 (SPSS Inc. Chicago, IL). Continuous variables were presented as means ± standard deviations or medians with interquartile ranges, and categorical variables were reported as counts and percentages. Shapiro–Wilk tests were used to test data normality. Normally distributed data were analyzed using the independent sample *t*-test; otherwise, the Wilcoxon rank-sum test was used. The chi-square test, and continuously correct chi-square test were used for categorical variables, as appropriate. We converted continuous variables into dichotomous variables by plotting the receiver-operating characteristic (ROC) curves and detecting the optimal discriminative threshold values. For univariate analysis, we included clinical, laboratory factors, and semi-quantitative, quantitative imaging factors with statistically significant between-group difference. Significant univariate predictors were then used for multivariate analysis to identify significant multivariate predictors. For multivariate analysis, we excluded parameters that showed non-significant differences between the two groups or had a too small number of events. Variables that had unconfirmed accuracy (e.g., myalgia, which was self-reported) or had collinearity with coexisting conditions were excluded. *P* < 0.05 was considered to indicate a statistical significance.

## Results

### Clinical Features of COVID-19 Pneumonia With and Without ICU Admission

Compared with patients without ICU admission, patients who were transferred to ICU were older (60.12 ± 14.29 vs. 40.50 ± 16.47, *P* < 0.001) and more likely to have coexisting conditions (52.50 vs. 19.34%, *P* < 0.001). However, there was no significant difference in exposure history to Wuhan or family cluster (Table 1). Cough, myalgia, and dyspnea occurred more frequently in patients with ICU admission, and these patients also had longer duration from onset of symptoms to hospital admission [4 days, (2, 6) vs. 1 day (1, 5), *P* = 0.044]. In addition, they were more likely to have higher systolic pressure, lower hemoglobin count, and lower lymphocyte count. D-dimer, alanine aminotransferase (ALT) and lactic dehydrogenase (LDH) were greater in patients with ICU admission than those without. Patients with ICU admission were more likely to have abnormal leucocyte count (>10 × 109/L), lymphocyte count (<0.8 × 109/L), APTT (>35 s), D-Dimer (>0.5 mg/L), ALT (>49 U/L), and LDH (>215 U/L). More results of laboratory findings were listed in Table 2.

**Table 1:** Demographics and baseline characteristics of patients with COVID-19 pneumonia.

	**All patients (*n* = 221)**	**ICU care (*n* = 40)**	**Non-ICU care (*n* = 181)**	***P*-value**
**Characteristics**				
Age, years	51.03 (17.20)	60.12 (14.29)	45.40 (16.47)	**<0.001**
**Age > 60 years old**				
Gender				0.103
Female	96 (43.44%)	22 (55.00%)	74 (40.88%)	
Male	125 (56.56%)	18 (45.00%)	107 (59.12%)	
Exposure history to Wuhan > 2 weeks	22 (9.95%)	3 (7.5%)	19 (10.5%)	0.567
Family cluster	28 (12.67%)	4 (10%)	24 (13.26%)	0.575
**Coexisting conditions**				
Any	66 (29.86%)	21 (52.50%)	35 (19.34%)	**<0.001**
Cardiovascular disease	39 (17.64%)	18 (45.00%)	21 (11.60%)	**<0.001**
Chronic obstructive pulmonary disease	7 (3.17%)	5 (12.50%)	2 (1.10%)	**<0.001**
Chronic liver disease	3 (1.36%)	3 (7.50%)	0	
Diabetes mellitus	8 (3.62%)	6 (15.00%)	2 (1.10%)	**<0.001**

*Data are number (%) or mean (SD). ICU, intensive care unit*.

*Bold font indicates statistical significance*.

**Table 2:** Onset of symptoms, vital signs, and laboratory findings in patients with COVID-19 pneumonia.

	**All patients**	**ICU care**	**Non-ICU care**	***P*-value**
	**(*n* = 221)**	**(*n* = 40)**	**(*n* = 181)**	
**Onset of symptoms**				
Fever	148 (66.97%)	30 (75.00%)	118 (65.19%)	0.233
Cough	82 (37.10%)	25 (62.50%)	57 (31.49%)	**<0.001**
Myalgia	40 (18.10%)	13 (32.50%)	27 (14.92%)	**0.015**
Headache	27 (12.22%)	5 (12.50%)	22 (12.15%)	0.952
Diarrhea	18 (8.14%)	5 (12.50%)	13 (7.18%)	0.266
Dyspnoea	37 (16.74%)	19 (47.50%)	18 (9.94%)	**<0.001**
Asymptomatic	4 (1.81%)	0	4 (2.21%)	-
**Vital signs**				
Duration from onset of symptoms to hospital admission, days	3 (1–6)	4 (2–6)	1 (1–5)	**0.044**
Highest temperature, °C				0.357
≤ 38	114 (51.58%)	18 (45.00%)	96 (53.04%)	
>38	107 (48.42%)	22 (55.00%)	85 (46.96%)	
Respiratory rate, breaths/min	20 (19, 22)	20 (19, 22)	20 (20, 24)	0.102
Systolic pressure, mm Hg	128 (120, 139)	132 (120, 143)	125 (119, 131)	**0.004**
**Laboratory findings**				
Leucocyte count, × 10^9^/L	4.90 (3.69, 6.12)	5.56 (4.02, 6.47)	4.66 (3.66, 5.96)	0.183
>10	8 (3.62%)	5 (12.50%)	3 (1.66%)	**0.001**
<4	68 (30.77%)	9 (22.50%)	59 (32.60%)	0.211
Neutrophils count, × 10^9^/L	3.50 (2.50, 4.24)	3.60 (2.53, 4.98)	3.45 (2.49, 4.11)	0.818
>6.30	8 (3.62%)	3 (7.50%)	5 (2.76%)	0.147
<1.80	26 (11.76%)	3 (7.50%)	23 (12.71%)	0.355
Lymphocyte count, × 10^9^/ L	1.20 (0.90, 1.70)	1.13 (0.73, 1.46)	1.28 (0.86, 1.80)	**0.030**
<0.80	30 (13.57%)	10 (25.00%)	20 (11.05%)	**0.020**
Hemoglobin count, g/L	130.23 (21.80)	108 (24.51)	133 (31.23)	**0.000**
<110	12 (5.43%)	1 (2.50%)	11 (6.08%)	0.366
Platelet count, × 10^9^/L	210.01 (74.40)	202.40 (69.15)	202.07 (62.31)	0.841
<100	6 (2.71%)	6 (15.00%)	0	—
Prothrombin time, s	12.3 (11.3, 13.6)	12.5 (11.4, 13.6)	12.1 (11.3, 13.0)	0.190
>16	35 (15.84%)	10 (25.00%)	25 (13.81%)	0.079
APTT, s	28.60 (26.00, 32.30)	29.0 (26.20, 33.10)	28.35 (26.15, 31.35)	0.564
>35	11 (4.98%)	5 (12.50%)	6 (3.31%)	**0.016**
D-Dimer, mg/L	0.46 (0.29, 0.88)	0.86 (0.37, 2.27)	0.36 (0.25, 0.60)	**<0.001**
>0.5	69 (31.22%)	23 (57.50%)	46 (25.41%)	**<0.001**
ESR, mm/H	28.00 (16.00, 45.75)	34.00 (16.50, 56.50)	28.50 (16.00, 37.25)	0.051
>15	89 (40.27%)	15 (37.50%)	74 (40.88%)	0.693
ALT, U/L	29.00 (22.15, 44.92)	33.26 (22.83, 46.56)	24.65 (19.95, 34.90)	**0.026**
>49	35 (15.84%)	11 (27.50%)	24 (13.26%)	**0.026**
AST, U/L	23.76 (19.04, 35.55)	23.05 (19.05, 29.63)	25.00 (19.00, 40.00)	0.491
>35	20 (9.05%)	3 (7.50%)	17 (9.39%)	0.706
Total bilirubin, (μmol/L)	13.12 (6.62)	12.10 (3.48)	11.85 (6.65)	0.255
>21	26 (11.76%)	5 (12.50%)	21 (11.60%)	0.873
Creatinine, μmol/L	78.21 (66.94)	92.60 (148.66)	74.91 (22.64)	0.154
>133	20 (9.05%)	4 (10.00%)	16 (8.84%)	0.817
LDH, U/L	215.95 (159.83, 313.25)	402.10 (218.50. 545.88)	208.60 (151.78, 246.50)	**0.003**
>215	77 (31.84%)	24 (60.00%)	53 (29.28)	**<0.001**
Oxygen saturation, %	95 (93, 97)	95 (93, 97)	96 (94, 97)	0.106

*Data are number (%) or median (IQR)*.

*ICU, intensive care unit; APTT, activated partial thromboplastin time; ESR, erythrocyte sedimentation rate; ALT, alanine aminotransferase; AST, aspartate transaminase; LDH, lactic dehydrogenase*.

*Bold font indicates statistical significance*.

### CT Findings Between ICU Care Group and Non-ICU Care Group

**Figure 4:**
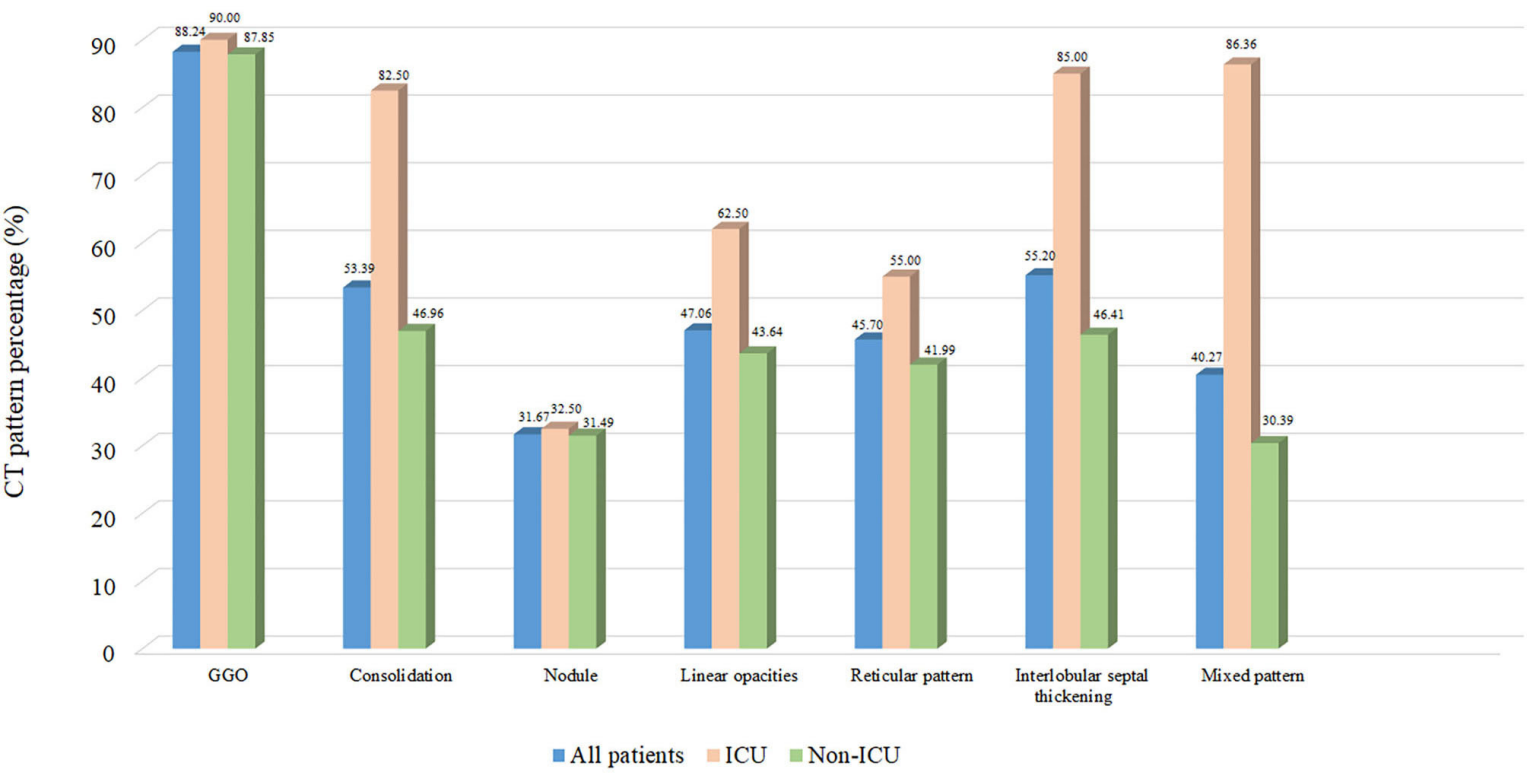
Distribution of various patterns in COVID-19 pneumonia.

Twenty patients showed no abnormality on chest CT imaging, and were all categorized into the non-ICU care group. Patients with adverse hospital outcomes showed a distribution pattern of diffuse and bilateral involvement more frequently than a distribution pattern of peripheral and unilateral involvement on admission. Forty-four patients (24.31%) in the non-ICU care group had only one lobe infected, patients in the ICU care group all showed an abnormality on chest CTs. Twenty-eight patients (70.00%) in the ICU care group had five lobes infected, whereas only 44 patients (24.31%) in the non-ICU care group showed involvement in five lobes. When interrogating each of the seven dominated image features, consolidation (82.50 vs. 46.96%, *P* < 0.001, linear opacity (62.50 vs. 43.64%, *P* = 0.031), interlobular septal thickening (85.00 vs. 46.41%, *P* < 0.001), and mixed pattern (86.36 vs. 30.39%, *P* = 0.000) showed a significantly higher occurrence rate in the ICU-care group than in the non-ICU care group. No significant differences were found in GGO (90.00 vs. 87.85%, *P* = 0.720), nodule (32.50 vs. 31.49%, *P* = 0.202), and reticular pattern (55.00 vs. 41.99%, *P* = 0.439) (Figure 4). The CT score of the ICU care group was significantly higher than that of the non-ICU care group (*P* = 0.003). Patients in the ICU care group tended to possess a higher rate of enlarged lymph node (short-axis diameter > 10 mm) compared with the non-ICU care group (22.73 vs. 3.87%, *P* < 0.001). Moreover, the coronary calcification occurrence rate was significantly higher in the ICU care group than in the non-ICU care group (31.82 vs. 5.36%, *P* < 0.001). Other extrapulmonary abnormalities, such as pleural effusion and aortic calcification based on chest CT, were identified more frequently in the ICU care group (10.00 vs. 3.31%, *P* = 0.066; 17.50 vs. 8.84%, *P* = 0.181), although the differences were not significant. All above comparisons were summarized in Table 3.

**Table 3:** CT findings between ICU group and non-ICU group.

	**All patients**	**ICU care**	**Non-ICU care**	***P*-value**
	**(*n* = 221)**	**(*n* = 40)**	**(*n* = 181)**	
Abnormal CT findings	201 (90.95%)	40 (100%)	161 (88.95%)	0.057
**Distribution of initial pulmonary lesions**				
Peripheral	110 (49.77%)	8 (20%)	102 (56.35%)	**<0.001**
Random	45 (20.36%)	12 (30%)	33 (18.23)	0.098
Diffuse	43 (19.46%)	16 (40.00%)	27 (14.92%)	**<0.001**
**Involvement of the lesions**				
Bilateral	127 (57.47%)	32 (80.00%)	95 (52.63%)	**0.001**
Unilateral	74 (33.48%)	8 (20.00%)	66 (36.46%)	0.046
**Number of the involvement lobes**				
One	44 (19.91)	0	44 (24.31%)	
Two	30 (13.57%)	8 (20.00%)	22 (12.15%)	0.120
Three	14 (6.25%)	2 (5.00%)	12 (6.63%)	0.190
Four	28 (12.67%)	2 (5.00%)	26 (14.36%)	0.107
Five	72 (32.58%)	28 (70.00%)	44 (24.31%)	**<0.001**
**CT findings of lung abnormalities evaluated by radiologists**				
GGO	195 (88.24%)	36 (90.00%)	159 (87.85%)	0.720
Consolidation	118 (53.39%)	33 (82.50%)	85 (46.96%)	**<0.001**
Nodule	70 (31.67%)	13 (32.50%)	57 (31.49%)	0.202
Linear opacities	104 (47.06%)	25 (62.50%)	79 (43.64%)	0.031
Reticular pattern	101 (45.70%)	22 (55.00%)	76 (41.99%)	0.134
Interlobular septal thickening	122 (55.20%)	34 (85.00%)	84 (46.41%)	**<0.001**
Mixed pattern	89 (40.27%)	34 (86.36%)	55 (30.39%)	**<0.001**
**Extrapulmonary abnormalities based on chest CT**				
Pleural effusion	10 (4.52%)	4 (10.00%)	6 (3.31%)	0.066
Lymph node (short axis diameter larger than 10 mm)	16 (7.24%)	9 (22.50%)	7 (3.87%)	**<0.001**
Coronary artery calcification	23 (10.41%)	13 (32.50%)	10 (5.52%)	**<0.001**
Aortic calcification	23 (10.41)	7 (17.50%)	16 (8.84%)	0.181
CT score	3 (1–7)	9 (5–13)	2 (1–5)	**0.003**
>7	19 (8.60%)	13 (32.5%)	6 (3.31%)	**<0.001**
**Quantitative index automatically calculated by DL software**				
TOP	2 (0.10, 13.05)	14 (8.00, 34.00)	9 (0.1–11.88)	**<0.001**
>10.5%	32 (14.48%)	21 (52.50%)	11 (6.08%)	**<0.001**
TOV (mm3)	87.29 (9.02, 463.25)	615.40 (305.40,1032.12)	615.4 (9.02–463.25)	**<0.001**
>150.72	40 (18.10%)	22 (55%)	18 (9.94%)	**<0.001**
TGP	0.2 (0.1, 2.30)	4.40 (2.10, 6.30)	0.65 (0.10, 2.55)	**<0.001**
>2.05%	19 (8.68%)	16 (40.00%)	13 (7.18%)	**<0.001**
TGV (mm3)	9.32 (0.77, 58.06)	69.55 (25.10, 175.27)	21.34 (0.82, 101.72)	**<0.001**
>24.59	34 (15.38%)	16 (40.00%)	18 (9.94%)	**<0.001**
TCP	1.05 (0, 8.48)	12.50 (6.40, 25.11)	0 (0, 1.53)	**<0.001**
>4.2%	31 (14.02%)	18 (45.00)	13 (7.18)	**<0.001**
TCV (mm3)	36.01 (0, 378.03)	398.00 (163.09, 854.98)	0 (0, 79.67)	**<0.001**
>139.47	39 (17.65%)	20 (50.00%)	19 (10.50%)	**<0.001**

*ICU, intensive care unit; GGO, ground-glass opacity; TOP, total opacity percentage; TOV, total opacity volume; TGP, total grand glass opacity percentage; TGV, total grand glass opacity volume; TCP, total consolidation percentage; TCV, total consolidation volume; AI, artificial intelligence*.

*Bold font indicates statistical significance*.

**Figure 5:**
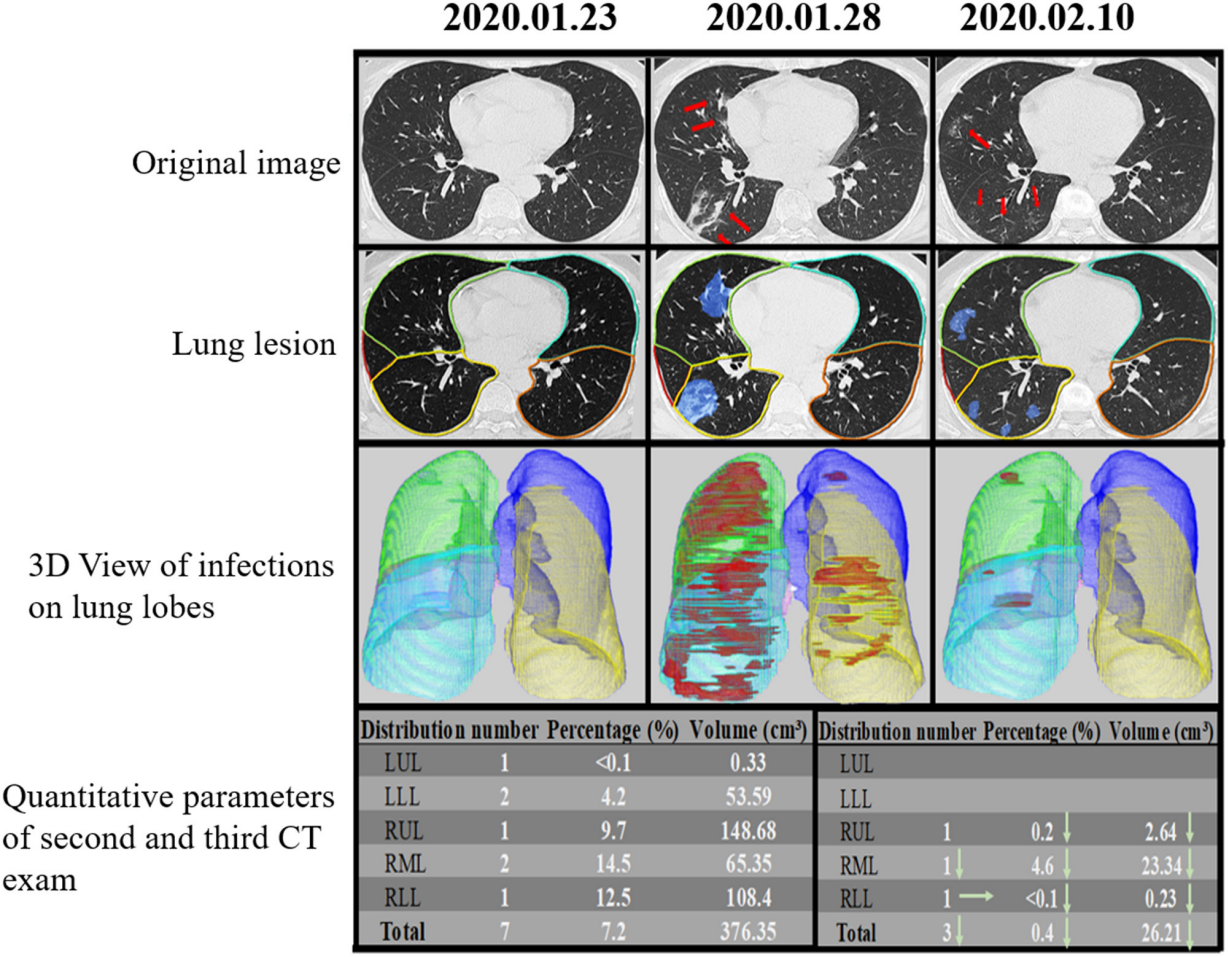
Temporal lung changes of a confirmed case in the non-ICU group on thin Slice CT. A 48-year-old man who was confirmed with COVID-19 pneumonia after fevered for 2 days. He was discharged after 18 days of treatment without ICU admission. Original image at three-time points (dates annotated above each panel) showed no abnormality at the first time, GGOs, and consolidations appear 6 days later, and lesions gradually absorbed 17 days later, only leaving some ill-defined GGOs. Lung lesion images were AI assist system at the same level and time points, showing the infection area in blue. 3D View of Infections on Lung Lobes was three-dimensional volume-rendered reconstructions at the same time points as in original green, RML in pink, RLL in blue. The quantitative parameters table showed the lesion percentage and volume in each lobe on the second and third scan. LUL, left upper lobe; LLL, left lower lobe; RUL, right upper lobe; RML, right middle lobe; RLL, right lower lobe.

**Figure 6:**
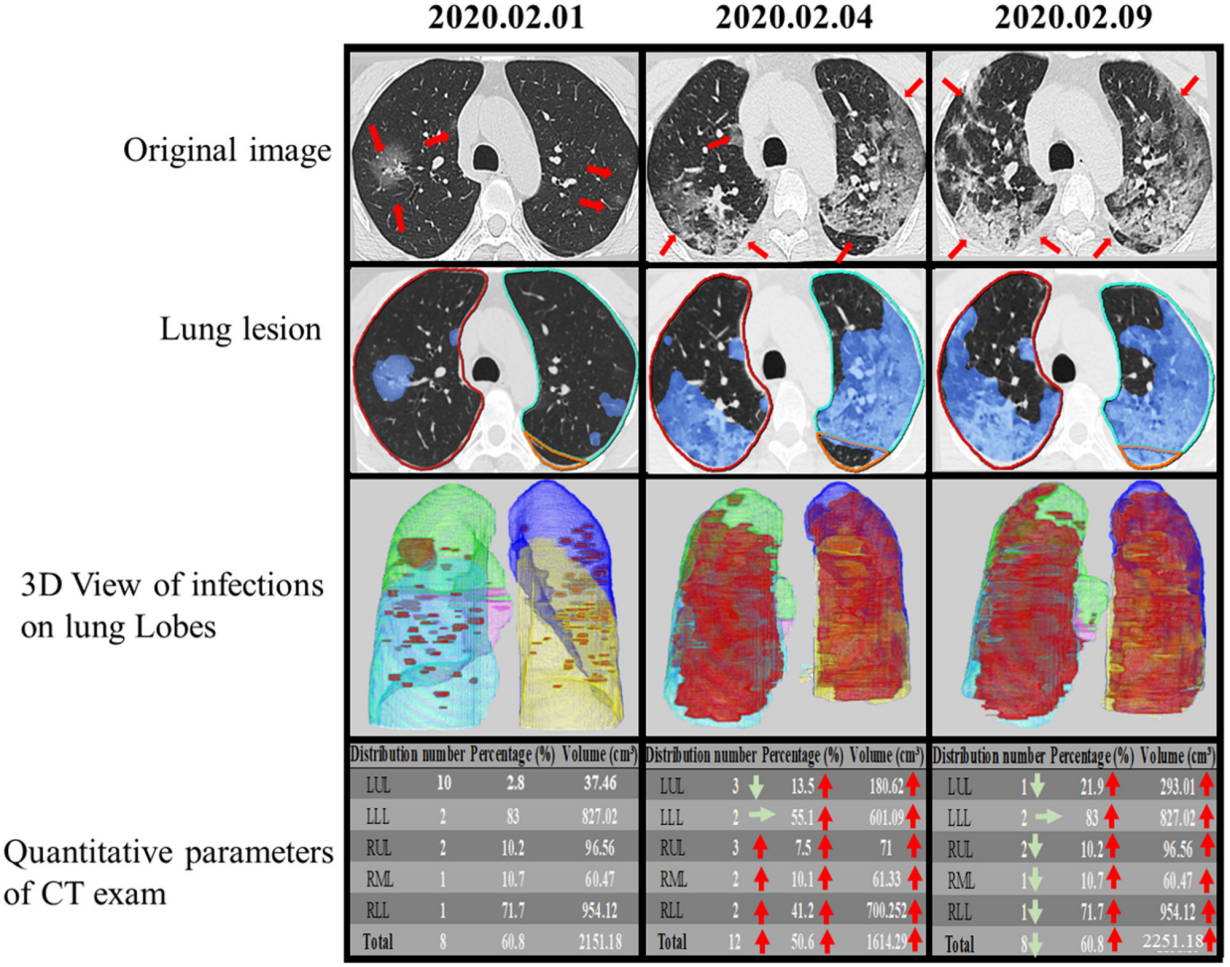
Temporal lung changes of one confirmed case in ICU group on thin slice CT. A 52-year-old man with a history of fever, cough, and myalgia for 3 days. Original images obtained on the 1st day, the 4th day, and the 9th day after admission. Multiple GGOs were distributed in both lungs on initial. Follow up exams showed rapid progress of the GGOs with the reticular pattern. Infection lesion Images colored infection area in blue. 3D View of Infections on Lung Lobes showed opacities displayed in red, LUL in purple, LLL in yellow, RUL in green, RML in pink, RLL in blue. The quantitative parameters table showed the lesion percentage and volume in each lobe. LUL, left upper lobe; LLL, left lower lobe; RUL, right upper lobe; RML, right middle lobe; RLL, right lower lobe.

**Figure 7:**
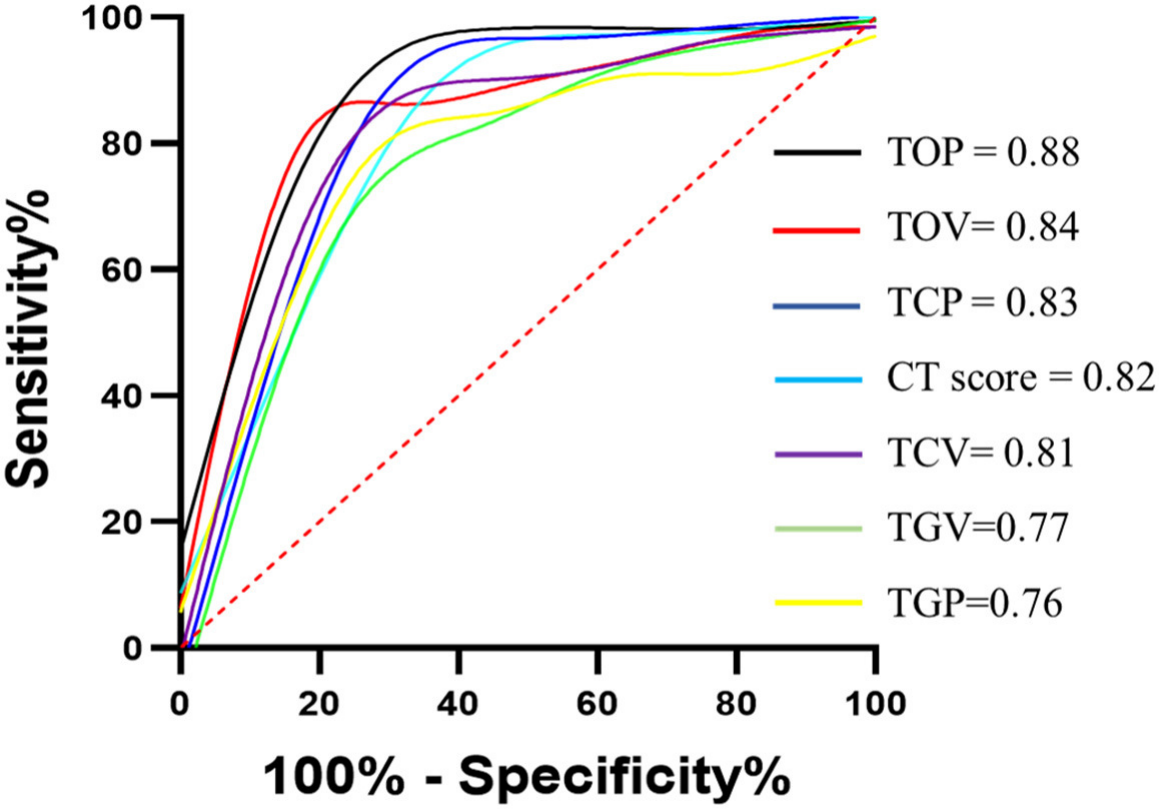
ROC curves for semiquantitative and quantitative parameters. ROC curves for CT score, TCV, TCP, TGV, TGP, TOV, and TOP. 95% CI for confidence intervals for area under curves (AUCs) were shown for CT score (0.73–0.91), TCV (0.74–0.90), TCP (0.74–0.92), TGV (0.66–0.86), TGP (0.67–0.86), TOV (0.77–0.92), and TOP (0.81–0.95). All *p* < 0.001. TCV, total consolidation volume; TCP, total consolidation percentage; TGV, total round glass opacity volume; TGP, total grand-glass opacity percent; TOV, total opacity volume; TOP, total opacity percentage.

The DL-based computer-aided diagnostic system revealed significant differences in CT score, TCV, TCP, TGV, TGP, TOV, and TOP (all *P* < 0.05) between the two groups (Table 3 and Figures 5, 6). The area under curves (AUCs) were 0.82 (95% confidence interval [CI], 0.73–0.91), 0.81 (95% CI, 0.74–0.90), 0.83 (95%CI, 0.66–0.86), 0.77 (95% CI, 0.74–0.92), 0.76 (95% CI, 0.66–0.86), 0.84 (95% CI, 0.77–0.92), and 0.88 (95% CI, 0.81–0.95), respectively, for these quantitative parameters, and optimal cutoff values were 7, 139.47 mm^3^, 4.2%, 24.59 mm^3^, 2.05%, 150.72 mm^3^, 10.5%, respectively (all *P* < 0.001; Figure 7).

### Risk Factors Associated With ICU Admission

**Figure 8:**
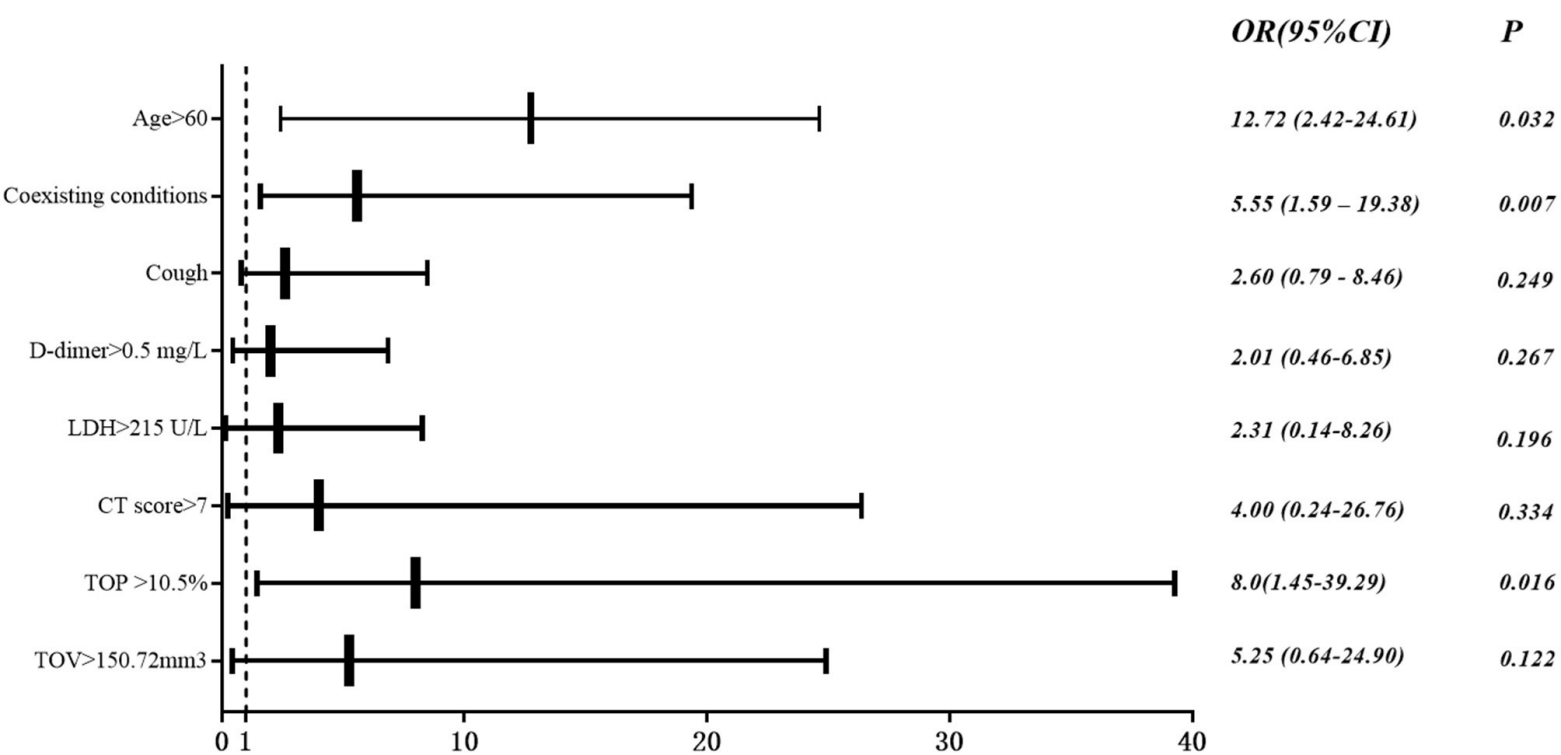
Multivariate logistic analysis. Odds ratio (OR) and 95% CI of ICU group vs. non-ICU group based on multivariable logistic regression for clinical and imaging features.

Univariate analysis showed age>60 years old, coexisting conditions, cough, myalgia, dyspnea, duration from onset of symptoms to hospital admission, systolic pressure, lymphocyte count (<0.8 × 109/L), ATPP (>35 s), D-dimer (<0.5 mg/L), LDH (>25 U/L), CT score (>7), TGP (>2.05%), TCP (>4.2%), TOV (>150.72 mm^3^), TGV (>2.4%) and TCV (>24.59 mm^3^) were the significant predictors for adverse outcomes. In multivariate analysis, age > 60 years old (odds ratio [OR], 12.72; 95% confidence interval [CI], 2.42–24.61; *P* = 0.032), coexisting conditions (OR, 5.55; 95% CI, 1.59–19.38; *P* = 0.007) and CT derived total opacity percentage (TOP) (OR, 8.0; 95% CI, 1.45–39.29; *P* = 0.016) remained significant (Table 4 and Figure 8).

**Table 4:** Univariate and multivariate analysis of clinical and imaging parameters for patients with ICU admission.

**Variables**	**Univariate**		**Multivariate**	
	**Odds ratio (95%CI)**	***P*-value**	**Odds ratio (95%CI)**	***P*-value**
Age > 60 years	10.13 (4.91–17.35)	**0.000**	12.72 (2.42–24.61)	**0.032**
Coexisting conditions	4.61 (2.24–9.49)	**0.010**	5.55 (1.59–19.38)	**0.007**
Cough	2.17 (1.08–4.39)	**0.030**	2.60 (0.79–8.46)	0.249
Dyspnoea	4.265 (1.03–18.57)	**0.000**	–	–
Duration from onset of symptoms to hospital admission, days	1.18 (1.02–1.37)	**0.028**	–	–
Systolic pressure	1.03 (1.01–1.05)	**0.004**	–	–
Lymphocyte count < 0.8 × 10^9^/L	2.68 (0.98–7.70)	0.055	–	–
Hemoglobin count, g/L	1.04 (1.02–1.06)	**0.000**	–	–
ATPP > 35 s	3.55 (1.55–27.65)	**0.010**	–	–
D-dimer > 0.5 mg/L	5.09 (1.73–14.93)	**0.003**	2.01 (0.46–6.85)	0.267
ALT > 49 U/L	2.25 (1.00–5.08)	**0.050**	–	–
LDH > 215 U/L	3.59 (1.77–7.31)	**0.000**	2.31 (0.14–8.26)	0.196
CT score > 7	14.09 (7.03–41.61)	**0.000**	4.00 (0.24–26.76)	0.334
TOP > 10.5%	17.08 (7.16–40.07)	**0.000**	8.0 (1.45–39.29)	**0.016**
TGP > 2.05%	8.62 (4.56–20.11)	**0.000**	–	–
TCP > 4.2%	10.57 (4.56–24.50)	**0.000**	–	–
TOV > 150.72 mm3	11.70 (5.02–24.40)	**0.000**	5.25 (0.64–24.90)	0.122
TGV > 2.4%	6.64 (2.72–13.41)	**0.009**	–	–
TCV > 24.59 mm3	8.53 (3.90–18.62)	**0.000**	–	–

*ICU, intensive care unit; APTT, activated partial thromboplastin time; ALT, alanine aminotransferase; LDH, lactic dehydrogenase; TOP, total opacity percentage; TGP, total grand glass opacity percentage; TCP, total consolidation percentage; TOV, total opacity volume; TGV, total grand glass opacity volume; TCV, total consolidation volume*.

*Bold font indicates statistical significance*.

## Discussion

The clinical characteristics of COVID-19 patients are diverse. Some patients have mild symptoms, while others develop severe and life-threatening symptoms. Early identification of common type patients with severe disease is beneficial to improve prognosis and reduce mortality. This study retrospectively analyzed the clinical and imaging data of COVID-19 patients hospitalized in multiple centers outside Wuhan and determined the clinical and imaging characteristics associated with subsequent transfer to ICU.

In this study, ICU admission occurred in 18.09% (40/221) of the patients after admission to hospital, which was significantly higher than the occurrence rate of 5.0% reported in Guan et al.'s (1) multicenter study, but substantially lower than 32% in Wuhan (24). These differential outcomes may be explained by the disparate health care resources across China. Most of the centers included in our study locate in the west and northeast of China, where medical resources were limited compared with the study of Guan (1) but are at a better level than the epicenter Wuhan. Among the 221 patients, only 3 (1.4%, 3/221) patients died during the hospitalization. The previous study described a different mortality rate among patients requiring ICU admission from 16% (25) to 38% (24), 62% (12), 67% (11), and 78% (26). In this study, the mortality rate was only 7.5% (3/40) that is significantly lower than those reported in previous studies, probably because of the difference in sample size, case inclusion criteria and the choice of treatment for the disease.

Compared with patients at risk of more severe illness, patients in the non-ICU care group were younger and possessed fewer coexisting conditions, which was in agreement with most studies regarding COVID-19 pneumonia. Up to 29.86% (66/21) patients in our non-ICU cohort had at least one comorbidity, which is significantly lower than Wang et al.'s study (25) (46.4%, 64/138) and Zhou et al.'s (26) study (48%, 91/191) but similar to Guan et al.'s study (1). Old age and basic diseases are the characteristics of susceptible groups of patients with acute and severe diseases. No differences existed in the proportion of men and women between ICU and non-ICU care groups, which may indicate that gender is not a risk factor for severe illness. Duration from the onset of symptoms to hospital admission was different in those two groups, emphasizing the importance of earlier screening and treatment. Among various onset symptoms in our cohort, only cough myalgia and dyspnea showed differences between the ICU and non-ICU care groups. Therefore, clinical recognition of the patients with such symptoms should be improved. It must be interpreted with the caution that while symptomatic patients tended to be mildly ill during hospitalization, they could transmit the virus to cohabiting family members and even cause severe COVID-19 pneumonia (27). In terms of laboratory tests, the absolute value of lymphocytes decreased in the ICU care group compared with the non-ICU care group, which may suggest that SARS-CoV-2 mainly acts on lymphocytes, especially T lymphocytes, as does SARS-CoV (28). ESR was higher in the ICU care group. Additionally, 5 (12.50%) patients in the ICU care group showed leucocyte count <10 × 10^9^/L, which is more frequent compared to the non-ICU care group (3, 1.66%). This may indicate that secondary bacterial infections have already occurred at the earlier stage and the inflammation plays an important role in the progression of the disease (29). A recent study showed that the virus may attack the heme on 1-beta chain of hemoglobin to dissociate the iron to form the porphyrin, which would impact hemoglobin from carrying oxygen and carbon dioxide (30). This may explain the decrease of hemoglobin in the ICU care group. We found that D-dimer was significantly higher in the ICU care group, implying the systemic pro-inflammatory cytokine response, the mediators of atherosclerosis, directly contributing to plaque rupture through local inflammation, induction of procoagulant factors, and hemodynamic changes, which predispose patients to ischemia and thrombosis (31). Higher alanine aminotransferase and lactic dehydrogenase may be associated with hepatic injury and indicate a poor prognosis as well (32).

Perhaps imaging could assist screening or accelerating the speed of diagnosis, especially with the shortage of reverse transcription-polymerase chain reaction assay (RT-PCR) in the epidemic center, such as Wuhan (33). Our study showed that 9.04% (20/221) of patients had negative lung parenchymal findings, and all of them were in the non-ICU care group. Thus, negative lung parenchymal may indicate a better prognosis. The previous article reported this clinic-radiological dissociation in COVID-19, pointing out that symptomatic patients were prone to having lung opacities (79%, 22/28) on CT, whereas only 54% (41/76) asymptomatic patients showed lung opacities on CT (34). The positive predictive value of CT will be low in the area where disease prevalence is low. Compared with the non-ICU care group, patients at risk for more severe illness may show diffused bilateral distribution patterns, including consolidation, linear opacity, interlobular septal thickening, and mixed pattern. Multiple consolidations may result from the filling of alveoli by inflammatory exudation, suggesting a more severe damage of alveoli and indicating a poor outcome (35). More involvement of opacifications is supposed to be associated with the immunopathological basis that coronavirus could interact with and modify the intracellular environment during infection for the benefit of quickly replicating (36). Since a dysregulated/exuberant innate response is the dominating factor to coronavirus-mediated pathology, it was of great importance to recognize high-risk imaging features that would result in deterioration on initial admission. We also focused on extrapulmonary abnormalities based on chest CT, finding that larger lymph nodes and coronary calcification also indicated poor outcomes, and those abnormalities could be detectable in the early stage of the disease. Enlarged lymph nodes may be resulted from severe inflammation (36). Coronary calcification may suggest the combination of CVD, which was reported as one of the most common comorbidities in critically ill patients (11).

We observed that compared with the non-ICU care group, the ICU care group showed a significantly higher value in the semi-quantitative parameter and quantitative parameter. At the same time, TOP achieved the best prediction for ICU admission (AUC = 0.88), followed by TOV (AUC = 0.84) and TCP (AUC = 0.83). We also found that TOP remained significant in multivariate analysis in the prediction of adverse outcomes. This independent predictive value may strongly suggest that the addition of total lung involvement may be the most valuable risk factor of COVID-19 pneumonia progression. A recent study conducted a multicenter retrospective study involving patients with moderate COVID-19 pneumonia to investigate the utility of CT and clinical characteristics to risk-stratify the patients and found that CT score was associated with inflammatory levels and that older age, higher neutrophil-to-lymphocyte ratio (NLR), and CT score on admission are independent risk factors for short-term progression (37). Similar to this research, we also found the CT score differences between groups, while its predictive value was surpassed by quantitative CT parameters.

Our data indicate that the presented multiparametric severity assessment (including biomarkers or imaging derived markers) showed excellent correlation with disease severity and individuals' risk for an aggravated course of disease. In clinical work, close monitoring and follow-up of risk indicators for early intensification and early intervention treatment will be possible to avoid the severity of COVID-19 and improve the prognosis of patients.

One noteworthy finding in this study is that the parameter measured by AI can be used for disease burden and disease progression prediction. In addition to rapid detection, DL-based technique can accurately evaluate lesion size, properties, and dynamic follow-up lesion alteration (18). Researchers have used AI in the precise segmentation of lesion regions and calculation of lesion volume, volume rates of lesions to total/left/right lung, and each lung lobe. Some researchers tried to apply AI in CT image analysis to differentiate COVID-19 from other viral pneumonia (38), or various community acquired pneumonia (39). A few research based on CT imaging focused on severity assessment of COVID-19, while most of them worked on distinguishing severe from non-severe patients. Recently, a deep learning-based survival model has been used in early triage of critically ill COVID-19 patients, however, this model lacked CT imaging features (40).

This study had several limitations. Firstly, some specific information from ICU, such as mechanical ventilation settings, therapeutic regimen, or extracorporeal membrane oxygenation (ECMO) were missing. Secondly, there have been many similar studies focused on COVID-19 pneumonia. However, since many countries still experiencing staggering daily increases of confirmed COVID-19 cases, different perspectives of studies might add some value to clinical judgment of uncontrolled COVID-19 pandemic. Thirdly, we didn't include cases from Wuhan city. However, since patients from Wuhan city have been fully explored, our study may help to cognize sporadic cases of COID-19. Fourth, although the commercial software has proven its accuracy in the quantitative evaluation of lung opacity, the current version still needs supervision or even manual correction by radiologists. In other words, the AI system needs further improvement in the segmentation accuracy of lung lobes and abnormalities.

## Conclusions

The older age, coexisting conditions and larger TOP were independently associated with ICU admission in patients with COVID-19 pneumonia. Early evaluation of risk factors for disease deterioration is warranted to control the progression of disease and implement appropriate therapies.

## Data Availability Statement

The original contributions presented in the study are included in the article/Supplementary Material, further inquiries can be directed to the corresponding author.

## Ethics Statement

The studies involving human participants were reviewed and approved by Ethics committee of Beijing Chaoyang Hospital. The patients/participants provided their written informed consent to participate in this study.

## Author Contributions

CY: conceptualization, formal analysis, investigation, data curation, writing–original draft, and writing–review and editing. YC: formal analysis, investigation, data curation, writing–original draft, and writing–review and editing. HY: formal analysis, investigation, and resource. JinX: methodology, software, and writing–review and editing. CH: methodology, software, and writing–review and editing. MY: software, writing–review and editing. YW, DW, TY, SW, ZL, FG, MK, WG, and QZ: resources. PS: investigation, data curation. XJ, ZF, and JiaX: writing–review and editing. SL and QY: conceptualization, writing–review and editing, resource, and supervision. All authors contributed to the article and approved the submitted version.

## Conflict of Interest

CH and JinX were employed by Beijing Deepwise & League of PHD Technology Co., Ltd. MY was employed by Neusoft Institute of Intelligent Healthcare Technology, Beijing, China. The remaining authors declare that the research was conducted in the absence of any commercial or financial relationships that could be construed as a potential conflict of interest.
