# Supplementary material for: Clinical Factors and Quantitative CT Parameters Associated With ICU Admission in Patients of COVID-19 Pneumonia: A Multicenter Study
Source: Front Public Health. 2021 Apr 22;9:648360. doi: 10.3389/fpubh.2021.648360 (PMC8101702; doi:10.3389/fpubh.2021.648360)
Supplement: Supplementary file 1 [file Presentation_1.pdf]

Supplementary Materials:

The input of the network was multiple CT slices, which forms the 3D input. The established U-Net [1] was used as the basic architecture for infection segmentation. To efficiently exploit 3D context information for auto-contouring of possible infection region, we replaced the 2D convolutional layers with 3D ones. Considering the overwhelming computational and memory consumption of normal 3D convolutional layers, we on the other hand simulate 3X3X3 3D convolution with 1X3X3 filter on the x-y plane plus 3X1X1 filter to build inter-slice connections on adjacent CT slices. Using such a pseudo-3D convolutional layers [2], the proposed model gained the ability of 3D context modeling while consuming much less memory and computational cost. Residual Unit could be given by: $X_{T+1} = h(X_T)+F(X_T)$, where X_T and X_{T+1} denote the input and output of the Tth Residual Unit, $h(X_T) = X_T$ is an identity mapping and F is a non-linear residual function.

We modified the basic Residual Unit in ResNet following the principle of Pseudo 3D as introduced aboved. The stacked architecture by making temporal 1D filters (*T*) follow spatial 2D filters (*S*) in a cascaded manner. Hence, the two kinds of filters could directly influence each other in the same path and only the temporal 1D filters are directly connected to the final output, which could be generally given by: $(I + T \cdot S) \cdot X_T := X_T + T(S(X_T)) = X_{T+1}$. The 3D ResNet-18 [3] architecture whose 3D convolutional layers were replaced with pseudo 3D convolutional layers was used as the backbone network for our proposed Pseudo 3D U-Net.

For the lung lesion segmentation, the Dice loss $Diceloss = 1 - \frac{2|X \cap Y|}{|X| + |Y|}$ and cross entropy loss $Crossentropyloss = -[ylog\hat{y} + (1-y)log(1-\hat{y})]$ were used. These two terms of loss function were given a weight of 0.5. For the lung lobe segmentation, the loss function here includes smooth edge loss and multi-class dice loss. The smooth edge loss was given by:

$$L_{sml}(X,Y) = \sum_{c=1}^{C}\sum_{i=1}^{D}\sum_{j=1}^{H}\sum_{k=1}^{W}\sum_{d\in\{x,y,z\}} m_d(X_c|i,j,k) \times w_d(Y_c|i,j,k)$$

$$m_d(X_c|i,j,k) = \begin{cases} |\partial_d(X_c|i,j,k) - \lambda| & , \partial_d(X_c|i,j,k) - \lambda > 0 \\ 0 & ,otherwise \end{cases}$$

$$w_d(Y_c|i,j,k) = e^{-|\partial_d(Y_C|i,j,k)|}$$

Where X and Y are the predicted probability map and the one-hot-encoding annotation of each lung lobe.

C was the category subscript, i,j,k was the voxel subscript, md (Xc|i,j,k) was the scalar edge loss calculated according to the gradient in the d direction. wd (Yc|i,j,k) was the weight mask that was used to eliminate the changes in the different categories. λ was the probability value of "mutation" in the segmentation result that may cause category jump.

The multi-class dice loss given by:

$$L_{lobes} = -2\sum_{c=0}^{5} \frac{\sum_{i=1}^{D}\sum_{j=1}^{H}\sum_{k=1}^{W} p_c^{(i,j,k)} \times q_c^{(i,j,k)}}{(\sum_{i=1}^{D}\sum_{j=1}^{H}\sum_{k=1}^{W} p_c^{(i,j,k)} + q_c^{(i,j,k)}) + \epsilon}$$

Where p and q are the predicted value and the encoded value by one-hot encoding respectively. i,j,k are the coordinate of the voxel, ε takes 1e-5 to prevent the denominator from being 0.

1. Ronneberger, O.; Fischer, P.; Brox, T. 2015. U-Net: Convolutional Networks for Biomedical Image Segmentation. https://arxiv.org/abs/1505.04597 (accessed on 18 May 2015)

2. Qiu, Z.; Yao, T.; Mei, T. Learning Spatio-Temporal Representation with Pseudo-3D Residual Networks. https://arxiv.org/abs/1711.10305 (accessed on 28 Nov 2017)

3. He, K.; Zhang, X.; Ren, S.; Sun, J. Deep Residual Learning for Image Recognition, 2016 IEEE Conference on Computer Vision and Pattern Recognition (CVPR), 2016, pp. 770-778. DOI: 10.1109/CVPR.2016.90
